# Supplementary material for: Inoculum of Endophytic Bacillus spp. Stimulates Growth of Ex Vitro Acclimatised Apple Plantlets
Source: Plants (Basel). 2025 Mar 27;14(7):1045. doi: 10.3390/plants14071045 (PMC11990893; doi:10.3390/plants14071045)
Supplement: Supplementary file 1 [file plants-14-01045-s001.zip › Supplementary-Materials.pdf]

Supplementary Materials

Inoculum of endophytic *Bacillus* spp. stimulates growth of ex vitro acclimatised apple plantlets

Jurgita Vinskienė [1], Inga Tamošiūnė [1], Elena Andriūnaitė [1], Dalia Gelvonauskienė [1], Rytis Rugienius [1], Muhammad Fahad Hakim [1], Vidmantas Stanys [1], Odeta Buzaitė [2], Danas Baniulis [1,*]

[1] Institute of Horticulture, Lithuanian Research Centre for Agriculture and Forestry, Kaunas Str. 30, 54333 Babtai, Kaunas reg., Lithuania; jurgita.vinskiene@lammc.lt (J.V.); inga.tamosiune@lammc.lt (I.T.); elena.andriunaite@lammc.lt (E.A.); rytis.rugienius@lammc.lt (R.R.); seteksna@gmail.com (D.G.); fahad.hakim@lammc.lt (M.F.H.); vidmantas.stanys@lammc.lt (V.S.);

[2] Department of Biochemistry, Vytautas Magnus University, Universiteto Str. 10, 53361 Akademija, Kaunas reg., Lithuania; odeta.buzaite@vdu.lt

* Correspondence: danas.baniulis@lammc.lt

Tables

Table S1. Summary statistics of metataxonomic analysis of apple tissue samples.

**Source**	**Buds**						**In vitro**			**CP**		
**Sample**	**B1**	**B2**	**B3**	**B4**	**B5**	**B6**	**IV1**	**IV2**	**IV3**	**CP1**	**CP2**	**CP3**
Total number of reads	76,206	163,354	100,786	80,417	30,162	128,151	103,268	90,067	147,950	68,046	73,966	28,899
Number of high-quality reads[a]	58,583	128,236	75,040	60,919	22,029	94,888	81,133	72,927	121,258	55,293	60,732	23,569
Number of mapped reads	32,045	69,639	45,396	34,534	13,229	55,870	47,538	42,874	64,558	31,599	32,270	13,327
Number of bacterial reads	15,293	33,391	28,889	19,544	8055	33,260	980	1084	1871	516	1164	341

Apple dormant bud (Buds), in vitro shoot (In vitro), and cryopreservation-treated in vitro shoot (CP) tissue samples were analysed using *16S rRNA* gene domain V4 amplicon high-throughput sequencing.

[a] filtered, denoised, and chimera-removed reads.

Table S2. Alpha diversity of endophytic bacteria in apple tissue samples.

Level	Index	Source			Hedge's g [CI 95%]		
		Buds	In vitro	CP	Buds – In vitro	Buds – CP	In vitro – CP
Family	Observed	19.5±1.64[a]	16.7±2.3[ab]	13.0±2.0[b]	1.4 [0.11, 3.9]	3.0 [1.8, 5.4]	1.1 [0, 2.2]
	Chao1	21.3±3.1[a]	17.0±2.7 [ab]	13.2±1.8[b]	1.3 [0.11, 6.4]	1.3 [1.7, 3.9]	0.9 [0.49, 1.4]
	ACE	24.0±5.0[a]	17.1±2.8[ab]	13.5±1.5[b]	1.2 [0.03, 6.6]	2.5 [1.6, 3.4]	1.5 [0.64, 2,4]
	Fisher's alpha	4.7±0.5[a]	3.8±0.7[ab]	2.8±0.5[b]	1.3 [0.13, 4.3]	3.1 [1.9, 5.1]	1.2 [0.16, 2.2]
	Shannon (H)	2.2±0.1[a]	2.0±0.2[ab]	1.7±0.5[b]	0.5 [0.88, 2.9]	0.9 [0.77, 3.3]	1.0 [0.28, 2.3]
	Simpson's (D)	0.85±0.01[a]	0.76±0.08[a]	0.69±0.19[a]	0.3 [1.4, 3.8]	0.7 [0.61, 4.2]	1.0 [0.51, 2]
Feature (ASV)	Observed	61.5±14.1[a]	19.0±3.5[b]	14.3±2.1[b]	3.6 [2.5, 8.8]	4 [2.9, 11]	1.2 [0.32, 7.8]
	Chao1	84.1±23.5[a]	19.3±3.8[b]	14.5±1.8[b]	3.3 [1.7, 6.2]	3.5 [2.0, 7.2]	1.2 [0.07, 8.3]
	ACE	87.8±28.2 [a]	19.5±3.9[b]	14.9±1.5[b]	2.9 [1.8, 5.0]	3.1 [1.8, 5.1]	1.1 [0.77, 8.0]
	Fisher's alpha	24.1±8.1[a]	4.5±1.1 [b]	3.2±0.6 [b]	2.9 [1.9, 6.8]	3.1 [2.2, 7.3]	1.2 [0.32, 8.3]
	Shannon (H)	3.4±0.3[a]	2.3±0.2[b]	2.0±0.5[b]	3.7 [2.5, 5.6]	2.8 [2.1, 3.5]	0.6 [0.82, 3.5]
	Simpson's (D)	0.94±0.02[a]	0.85±0.03[ab]	0.77±0.15[b]	2.6 [1.5, 9.0]	0.9 [0.78, 1.4]	0.5 [2.7, 17.0]

Family and feature (amplicon sequence variant (ASV)) level alpha diversity indices of apple dormant bud (Buds), in vitro shoot (In vitro), and cryopreservation-treated in vitro shoot (CP) tissue samples were estimated using *16S rRNA* domain V4 amplicon high-throughput sequencing data. Data presented as mean and standard deviation. The same letters denote no significant difference between the experimental groups ($p \leq 0.05$). Between-group differences are shown as the Hedge's g standardised mean difference effect size with a 95% confidence interval (CI 95%).

Table S3. Relative abundance of family-level taxa in apple tissue samples.

**Source**			**Buds**					**In vitro**			**CP**	
**Samples**	B1	B2	B3	B4	B5	B6	IV1	IV2	IV3	CP1	CP2	CP3
Enterobacteriaceae	-	-	-	-	-	-	43.8	54.8	34.1	35.1	71.9	40.1
Sphingomonadaceae	32.4	25.4	28.4	27.1	28.4	29.4	2.3	-	1.3	1.3	-	-
Beijerinckiaceae	13.0	24.4	11.4	13.4	13.4	22.7	0.3	-	1.0	0.3	-	0.7
Pseudomonadaceae	15.4	8.0	7.7	19.4	13.0	4.3	0.7	6.4	0.7	1.3	7.0	9.0
Bacillaceae	-	0.3	-	-	-	-	13.0	9.7	16.1	20.1	5.7	15.1
Lactobacillaceae	-	-	-	-	-	-	7.0	7.7	11.4	16.1	5.7	9.7
Oxalobacteraceae	5.7	6.0	3.0	10.0	13.0	10.0	1.7	-	1.3	1.0	-	-
Sphingobacteriaceae	10.7	6.0	13.0	4.3	4.7	7.4	-	-	-	-	-	-
Microbacteriaceae	7.4	7.4	5.4	5.0	5.7	5.4	0.3	-	4.7	-	-	-
Erwiniaceae	0.7	3.0	4.7	1.0	-	0.7	7.0	2.3	3.0	4.0	-	5.0
Neisseriaceae	-	-	-	-	-	0.3	8.7	4.0	5.0	4.7	2.7	4.3
Nocardiaceae	0.7	-	3.0	9.7	8.7	-	-	-	-	-	-	-
Moraxellaceae	-	-	-	-	-	-	2.3	0.7	8.7	4.0	2.3	2.0
Acetobacteraceae	2.3	6.0	5.0	1.7	1.0	2.0	-	-	-	-	-	-
Mycobacteriaceae	-	-	-	-	-	-	5.0	2.0	1.7	4.7	0.7	2.7
Comamonadaceae	1.7	2.0	6.0	2.0	1.0	4.0	-	-	-	-	-	-
Hymenobacteraceae	2.0	4.0	2.3	1.7	3.0	1.7	-	-	-	-	-	-
Micrococcaceae	-	-	-	-	-	-	0.3	3.7	3.0	2.3	0.3	5.0
Kineosporiaceae	1.0	2.3	2.0	2.0	4.0	2.3	-	-	-	-	-	-
Propionibacteriaceae	0.3	-	0.3	0.3	1.0	-	1.7	2.0	3.3	-	-	2.7

Table S3. Relative abundance of family-level taxa in apple tissue samples (continued).

**Source**			**Buds**					**In vitro**			**CP**	
**Samples**	B1	B2	B3	B4	B5	B6	IV1	IV2	IV3	CP1	CP2	CP3
Streptococcaceae	-	-	-	-	-	-	1.7	2.3	0.7	2.0	0.3	2.0
Abditibacteriaceae	1.0	1.0	2.0	0.3	0.3	3.7	-	-	-	-	-	-
Staphylococcaceae	-	-	-	-	-	-	2.0	1.3	2.7	1.0	1.0	-
Spirosomaceae	2.0	0.7	2.0	-	-	2.7	-	-	-	-	-	-
Xanthomonadaceae	-	-	-	-	-	-	-	1.3	0.7	-	2.3	1.7
Rhizobiaceae	1.0	0.7	1.0	0.3	0.3	1.0	-	-	-	-	-	-
Nocardioidaceae	0.7	-	2.3	0.3	-	-	-	-	-	-	-	-
Deinococcaceae	0.3	0.7	-	0.3	1.3	-	-	-	-	-	-	-
Geodermatophilaceae	1.0	0.7	-	-	0.3	0.3	-	-	-	-	-	-
Exiguobacteraceae	-	-	-	-	-	-	1.3	-	-	-	-	-
Amoebophilaceae	0.3	0.3	-	0.3	-	-	-	-	-	-	-	-
Solirubrobacteraceae	0.3	-	-	0.3	-	0.3	-	-	-	-	-	-
Chitinophagaceae	-	-	-	-	-	0.7	-	-	-	-	-	-
Diplorickettsiaceae	-	-	-	-	0.7	-	-	-	-	-	-	-
Burkholderiaceae	-	0.3	-	-	-	0.3	-	-	-	-	-	-
Ilumatobacteraceae	-	-	-	0.3	-	-	-	-	-	-	-	-
Nakamurellaceae	-	-	-	-	-	0.3	-	-	-	-	-	-
not assigned	-	-	-	-	-	-	0.7	1.7	0.7	2.0	-	-

Samples of dormant buds (Buds), in vitro shoots (In vitro), and cryopreservation-treated in vitro shoots (CP) were analysed using *16S rRNA* domain V4 amplicon high-throughput sequencing. The data were mapped using the SILVA SSU database. Abundance values higher than 5% and 20% are shown in blue and red font, respectively.

Table S4. Endophytic bacteria inoculum effect on apple in vitro propagated shoot rooting efficiency and ex vitro acclimatised plantlet growth parameters.

Experimental group	Rooting efficiency	Acclima-tisation efficiency	Cumulative root length, cm	Root number	Root length, cm	Leaf area, cm^2	Fresh weight, mg
Control	73±12[a]	90±13[a]	4.9±2.7[c]	4.1±1.7[b]	1.2±0.66[b]	1.4±0.68[b]	81.8±31.3[b]
Peribacillus frigoritolerans S1.2	74±14[a]	79±20[a]	5.1±3.1[bc]<br>0.06 [-0.29, 0.43]	4.1±1.8[b]<br>0.02 [-0.40, 0.41]	1.2±0.70[b]<br>0.07 [-0.12, 0.24]	1.4±0.70[b]<br>-0.06 [-0.45, 0.32]	86.8±32.9[b]<br>0.15[-0.21, 0.52]
Bacillus toyonensis S1.4	72±6[a]	87±12[a]	5.2±2.6[bc]<br>0.13 [-0.27, 0.51]	4.1±1.8[b]<br>0.01 [-0.34, 0.44]	1.2±0.69[b]<br>0.05 [-0.12, 0.23]	1.4±0.68[b]<br>-0.00 [-0.35, 0.38]	80.6±38.1[b]<br>-0.04 [-0.40, 0.333]
Bacillus sp. L3.4	84±12[a]	97±3[a]	7.5±4.8[a]<br>0.69 [0.40, 0.94]	5.4±2.8[a]<br>0.56 [0.24, 0.87]	1.3±0.65[a]<br>0.21 [0.08, 0.36]	2.1±1.1[a]<br>0.80 [0.49, 1.10]	112.0±48.2[a]<br>0.75 [0.45, 1.13]
Bacillus toyonensis Nt18	84±15[a]	91±15[a]	6.7±4.2[ab]<br>0.50 [0.05, 0.92]	5.1±2.9[ab]<br>0.44 [-0.02, 0.87]	1.3±0.69[ab]<br>0.16 [-0.02, 0.35]	2.4±1.3[a]<br>0.88 [0.44, 1.3]	127.0±59.3[a]<br>0.96 [0.52, 1.4]
Combined inoculum[1]	76±11[a]	92±11[a]	7.8±3.3[a]<br>0.96 [0.50, 1.4]	5.7±2.3[a]<br>0.78 [0.34, 1.2]	1.3±0.61[a]<br>0.25 [0.09, 0.45]	1.9±0.95[a]<br>0.61 [0.21, 0.98]	121.0±59.7[a]<br>0.83 [0.35, 1.2]

[1] The combined inoculum consisted of all four strains at four-fold lower concentration. The data are presented as mean and standard deviation. The same letters denote no significant difference between the experimental groups ($p \leq 0.05$). Between-group differences are shown as the Hedge's g standardised mean difference effect size with a 95% confidence interval (CI 95%).

Table S5. Summary statistics of metataxonomic analysis of ex vitro acclimated apple plantlet root samples.

**Treatment**	**Control**			***Bacillus* sp. L3.4**			***B. toyonensis* Nt18**			**Combined inoculum**		
**Samples**	C1	C2	C3	L1	L2	L3	N1	N2	N3	CI1	CI2	CI3
Total number of reads	219,514	101,352	226,764	191,630	297,410	159,810	218,882	166,866	93,781	114,995	202,930	140,902
Number of high-quality reads[a]	169,705	85,335	198,473	144,940	225,984	129,269	170,070	132,465	74,682	93,184	149,918	110,579
Number of mapped reads	169,685	85,317	198,399	144,900	225,799	129,142	170,025	132,433	74,652	93,159	149,883	110,554
Number of bacterial reads	54,945	41,502	71,076	58,007	85,458	49,573	67,824	52,078	29,498	48,299	52,223	39,058

Control and bacterial inoculum-treated samples were analysed using *16S rRNA* gene domain V4 amplicon high-throughput sequencing.
[a] filtered, denoised, and chimera-removed reads.

Table S6. Alpha diversity of bacteria in ex vitro acclimated apple plantlet root samples.

**Index**	**Treatment**				**Hedge's g [CI 95%]**		
	**Control**	***Bacillus* sp. L3.4**	***B. toyonensis* Nt18**	**Combined inoculum**	**Control – *Bacillus* sp. L3.4**	**Control – *B. toyonensis* Nt18**	**Control – Combined inoculum**
Observed	192±27[a]	135±45[ab]	91±21[ab]	118±21 [b]	1.2 [0.17, 16.2]	3.3 [2.3, 5.0]	2.4 [1.5, 96.7]
Chao1	192±27[a]	135±45 [ab]	92±22 [ab]	118±20[b]	1.2 [0.24, 21.9]	3.2 [2.3, 6.6]	2.4 [1.5, 63.0]
ACE	193±27[a]	135±44[ab]	92±22[ab]	119±20[b]	1.2 [0.18, 15.5]	3.2 [2.2, 6.6]	2.4 [1.5, 55.0]
Fisher's alpha	27.6±4.5[a]	18.4±7.1[ab]	11.7±3.0[ab]	15.8±3.2[b]	1.2 [0.17, 17.1]	3.2 [2.2, 3.8]	2.3 [1.4, 93.1]
Shannon (H)	3.6±0.2[a]	2.6±0.7[ab]	2.2±0.1[ab]	2.7±0.5[b]	1.1 [0.41, 29.7]	7.7 [6.3, 10.3]	1.4 [0.70, 22.3]
Simpson's (D)	0.94±0.14[a]	0.77±0.08[a]	0.72±0.00[a]	0.84±0.09[a]	1.0 [0.26, 16.1]	12.3 [11.2, 361.6]	1.0 [0.20, 44.1]

Family level alpha diversity indices were estimated using *16S rRNA* domain V4 amplicon high-throughput sequencing data. Data presented as mean and standard deviation. The same letters denote no significant difference between the experimental groups ($p \leq 0.05$). Between-group differences are shown as the Hedge's g standardised mean difference effect size with a 95% confidence interval (CI 95%).

Table S7. Relative abundance of family-level taxa in ex vitro acclimated apple plantlet roots.

**Samples**	**Control**			***Bacillus* sp. L3.4**			***B. toyonensis* Nt18**			**Combined inoculum**		
**Samples**	C1	C2	C3	L1	L2	L3	N1	N2	N3	CI1	CI2	CI3
Burkholderiaceae	25.2	6.4	10.0	11.3	63.3	60.8	54.6	55.2	50.7	9.2	41.9	38.6
Caulobacteraceae	17.2	27.3	28.3	20.6	3.8	3.3	14.1	13.0	25.1	26.0	10.3	7.0
Rhizobiaceae	5.9	8.2	8.3	11.7	6.5	8.9	1.7	1.5	1.0	11.6	3.5	4.1
Sphingomonadaceae	11.0	1.4	1.9	3.2	6.2	6.6	8.7	7.9	5.1	3.1	6.7	6.2
Yersiniaceae	-	-	0.3	0.1	1.1	1.7	0.7	0.3	0.3	-	25.3	30.7
Oxalobacteraceae	6.9	8.0	14.2	6.6	1.2	1.3	3.8	5.4	4.4	5.1	1.0	1.8
Comamonadaceae	10.8	2.6	1.3	18.4	0.2	0.2	0.9	1.2	0.5	14.0	0.5	1.1
Acidobacteriaceae (Subgr.1)	2.5	0.2	0.4	0.4	6.4	6.3	3.6	3.7	2.9	0.4	4.1	3.0
Pseudomonadaceae	2.8	16.3	11.5	-	-	-	0.2	-	0.1	-	-	-
Microbacteriaceae	1.1	0.2	0.1	0.3	5.1	4.2	4.1	4.4	3.2	0.2	1.5	1.4
Sphingobacteriaceae	1.8	4.0	2.2	3.0	2.5	2.4	1.7	1.6	1.3	1.6	0.7	0.8
Rhodanobacteraceae	2.4	5.6	2.2	2.9	0.3	0.5	0.3	0.3	0.4	3.6	0.3	0.7
Xanthomonadaceae	0.5	0.7	0.7	0.2	0.3	0.3	0.1	-	0.4	8.2	0.1	0.6
Beijerinckiaceae	2.4	0.5	0.9	2.2	0.7	0.7	0.6	0.5	0.5	0.8	0.8	0.8
Xanthobacteraceae	1.1	0.8	1.0	2.1	0.2	0.4	0.4	0.3	0.4	1.0	0.2	0.2
Nocardioidaceae	0.9	0.4	0.8	1.2	0.6	0.6	0.4	0.5	0.6	1.0	0.3	0.2
Azospirillaceae	1.1	2.1	1.1	0.6	-	-	-	-	-	1.7	0.5	0.2
Alcaligenaceae	0.4	0.9	0.7	0.6	0.3	0.5	0.3	0.5	0.3	1.1	0.4	0.5
Weeksellaceae	-	-	-	0.1	-	-	-	-	-	5.7	-	0.3
CPla 3 termite group	0.1	0.8	1.9	2.9	-	-	-	-	-	0.3	-	-
Bacillaceae	0.6	0.2	0.6	0.7	0.3	0.2	0.6	0.9	0.6	0.3	0.3	0.2
Acetobacteraceae	-	0.1	0.1	-	0.1	0.2	1.4	1.3	1.5	-	0.2	0.3

Table S7. Relative abundance of family-level taxa in ex vitro acclimated apple plantlet roots (continued).

**Samples**	**Control**			***Bacillus* sp. L3.4**			***B. toyonensis* Nt18**			**Combined inoculum**		
**Samples**	C1	C2	C3	L1	L2	L3	N1	N2	N3	CI1	CI2	CI3
Tepidisphaeraceae	0.5	0.5	0.7	2.0	-	-	-	-	-	0.3	-	-
Enterobacteriaceae	-	2.5	0.6	-	-	-	0.4	0.3	0.1	-	-	-
Spirosomaceae	0.4	0.9	0.6	1.0	-	-	-	-	-	0.6	-	-
Fimbriimonadaceae	0.1	0.5	0.5	1.6	-	-	-	-	-	0.2	-	-
Hyphomicrobiaceae	0.2	0.4	0.5	0.7	0.1	0.1	0.1	-	-	0.5	0.1	0.1
Sporolactobacillaceae	0.5	-	0.1	0.1	0.2	0.2	0.6	0.7	0.2	-	-	0.2
Erwiniaceae	0.3	1.5	0.9	-	-	-	-	-	-	-	-	-
Paenibacillaceae	0.4	0.3	0.4	0.4	0.1	0.1	-	-	-	1.0	-	-
Opitutaceae	0.1	-	1.8	0.4	-	-	-	-	-	0.2	-	-
Gemmataceae	0.1	0.8	0.4	0.4	-	-	-	-	-	0.3	-	-
Pedosphaeraceae	0.1	0.2	0.5	0.9	-	-	-	-	-	0.2	-	-
Mycobacteriaceae	0.3	-	-	0.1	0.2	0.2	0.1	-	0.1	0.1	0.3	0.3
Chitinophagaceae	0.2	0.7	0.5	0.1	-	-	-	-	-	0.1	-	-
Streptomycetaceae	-	0.8	0.5	0.1	-	-	0.1	-	-	0.1	-	-
WD2101 soil group	0.2	0.3	0.3	0.4	-	-	-	-	-	0.3	-	-
Methylophilaceae	0.4	0.3	0.3	0.2	-	-	-	-	-	0.2	-	-
Devosiaceae	0.1	0.2	0.2	0.3	-	-	-	-	-	0.3	0.1	-
Chthoniobacteraceae	-	0.7	0.2	-	-	-	-	-	-	-	-	-
Paracaedibacteraceae	-	0.1	-	-	0.1	0.1	-	-	-	0.1	0.3	0.1
Alicyclobacillaceae	-	-	0.1	0.1	-	0.1	0.1	0.2	0.1	-	-	-
not assigned	0.5	2.1	1.3	1.4	-	-	0.1	0.1	0.1	0.4	-	-

Control and bacterial inoculum-treated samples were analysed using *16S rRNA* domain V4 amplicon high-throughput sequencing. The data were mapped using the SILVA SSU database. Abundance values higher than 5% and 20% are shown in blue and red font, respectively.

Table S8. Statistics of metagenomic analysis of substrate samples collected from the root zone of ex vitro acclimated apple plantlets.

**Experimental group**	**Control**			**Combined inoculum**		
**Sample**	C1	C2	C3	T1	T2	T3
Sequencing data						
Clean data (bp)	6,961,598,751	7,427,681,832	7,329,272,263	7,519,588,055	7,440,789,996	7,445,635,209
No host data (bp)	6,287,947,346	6,684,035,347	6,520,023,142	6,742,141,243	6,716,293,527	6,662,745,120
Number of reads	42,027,040	44,700,532	43,601,106	45,089,272	44,919,622	44,537,970
GC (%)	60.6	61.4	60.9	63.2	60.8	64.8
Q30 (%)	93.1	93.0	92.8	92.2	93.0	92.2
Metagenome assembly						
Contig number	602,552	671,914	676,6	677,164	652,714	702,935
Total length (bp)	451,022,513	551,098,854	558,643,674	557,310,496	573,720,412	578,912,063
Largest length (bp)	1,177,444	832,736	595,526	459,372	1,057,958	429,693
N50 (bp)	789	936	954	967	1,073	959
GC (%)	60.8	61.7	61.3	63.4	61.3	65.1
Mapped (%)	69.1	79.3	77.9	75.0	76.4	77.8
Gene prediction						
Gene number	802,017	922,199	935,856	943,666	929,874	987,908
Total length (bp)	372,280,836	448,758,990	457,224,459	460,007,703	472,197,678	483,185,064
Average length (bp)	464	486	488	487	507	489

Control and combined bacterial inoculum-treated samples were analysed using shotgun high-throughput sequencing.
Note: Clean data (bp): clean data yield after QC; No host data (bp): data after host sequence removal; Number of reads: Counts of final valid reads; Q30 (%): percentage of bases with Q-score no less than Q30; N50 (bp): sort contigs from long to short and count the cumulative length, when a contig is added and the cumulative length equals half of the total lengths of all contigs, the length of this contig is defined as N50; GC (%): GC content, i.e., the percentage of G or C in total bases; Mapped (%): mapping ratio of sequencing reads against assembled contigs.

Table S9. Alpha diversity of bacteria in substrate samples collected from the root zone of ex vitro acclimated apple plantlets.

**Index**	**Treatment**		**Hedge's g [CI 95%]**
	**Control**	**Combined inoculum**	**Control – Combined inoculum**
Observed	14304±119[a]	13714±787[a]	0.61 [-1.4, 10.0]
Chao1	14314±119[a]	13727±785[a]	0.61 [-1.9, 8.3]
ACE	14328±120[a]	13742±782[a]	0.61 [-1.9, 8.3]
Shannon (H)	6.28±0.08[a]	6.30±0.04[a]	-0.26 [-3.0, 3.5]
Simpson's (D)	0.9895±0.0005[a]	0.9916±0.0003[b]	-3.5 [-2.1, -14.5]

Family level alpha diversity indices were estimated using shotgun metagenomic sequencing data. Data presented as mean and standard deviation. The same letters denote no significant difference between the experimental groups ($p \leq 0.05$). Between-group differences are shown as the Hedge's g standardised mean difference effect size with a 95% confidence interval (CI 95%).

Tables provided in a separate MS Excel file:

Table S10. Relative abundance of genus-level taxa in the root zone of ex vitro acclimated apple saplings.
Table S11. Metabolic pathways including differential abundance genes identified using KEGG database.
Table S12. KO terms of differential abundance genes identified using KEGG database.
Table S13. Results of gene abundance analysis using NCycDB database.
Table S14. Results of gene abundance analysis using PCycDB database.

Figures

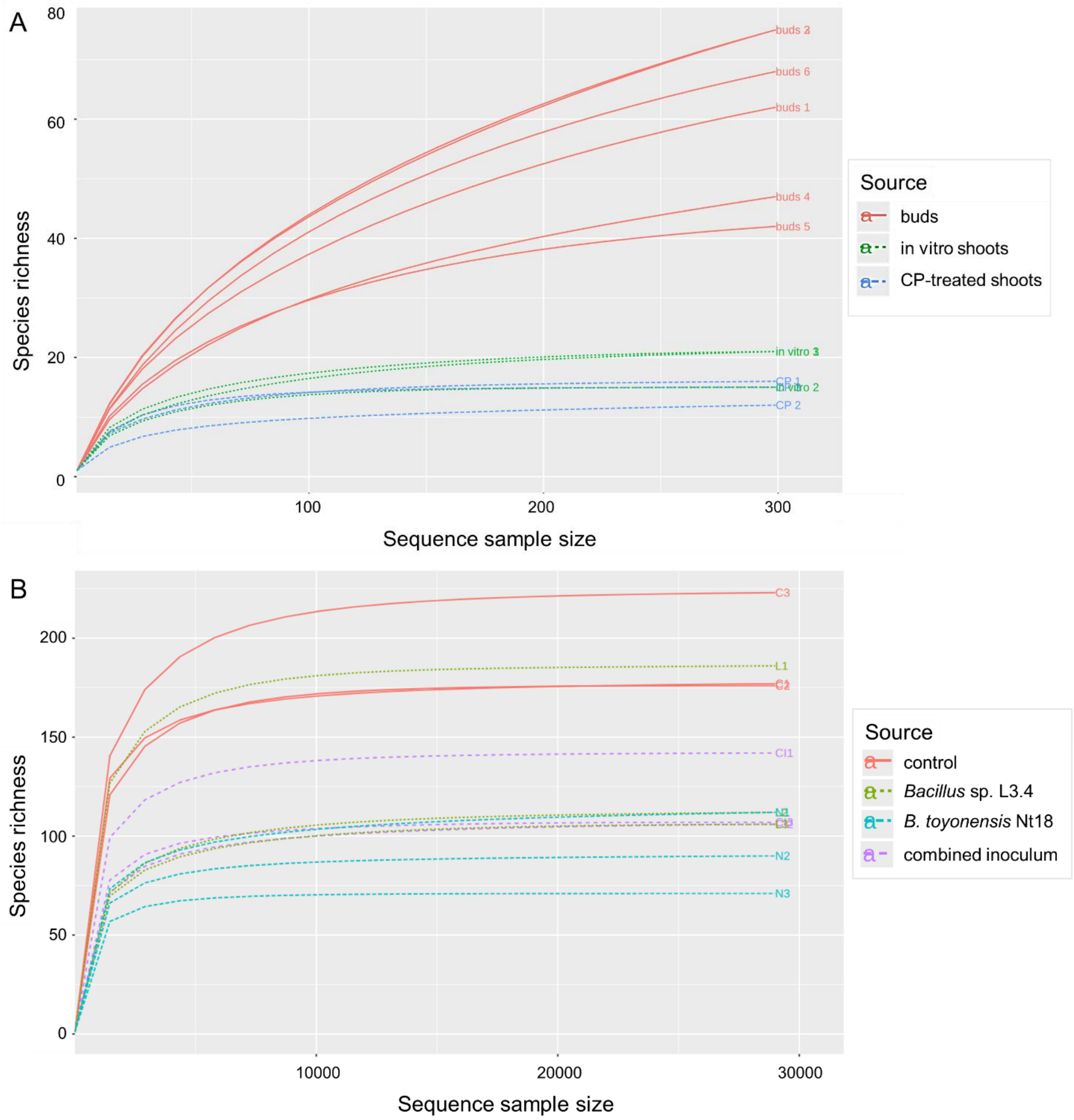

Figure S1. Rarefaction curves of apple tissue and ex vitro acclimatised plantlet root samples. Rarefaction curves for apple dormant bud, in vitro shoot, and cryopreservation-treated in vitro shoot (CP-treated shoots) tissue samples (**A**) and control and bacterial inoculum-treated ex vitro acclimatised plantlet root samples (**B**) were estimated from *16S rRNA* domain V4 amplicon high-throughput sequencing data.

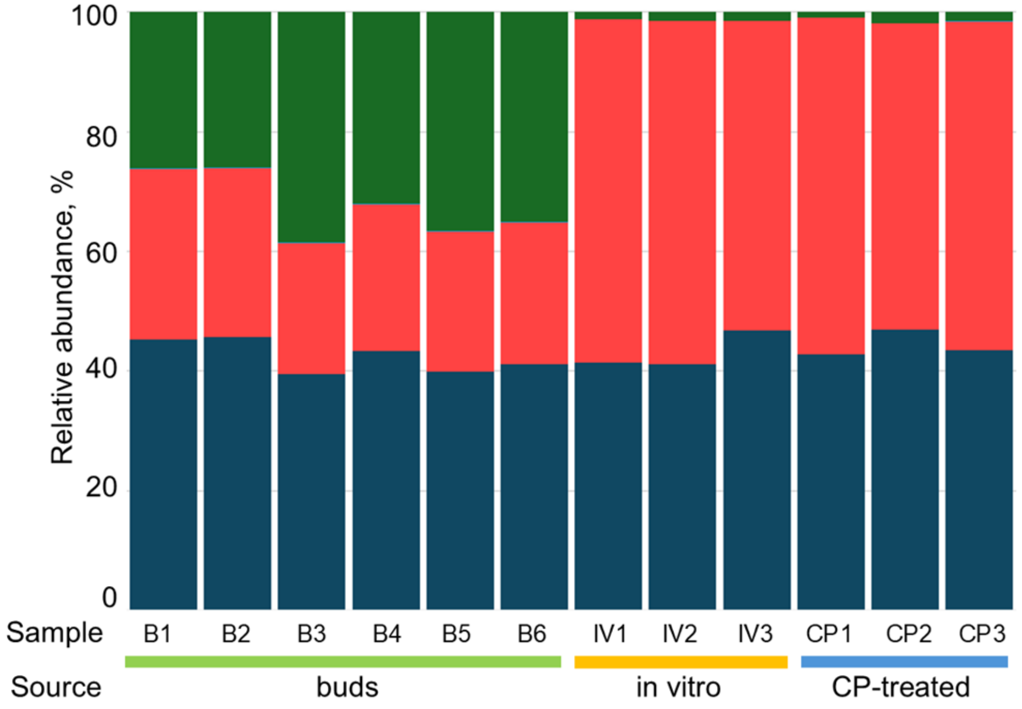

Figure S2. Distribution of the bacterial (**green**), mitochondrial (**red**), and not assigned (**blue**) reads. Data obtained from *16S rRNA* domain V4 amplicon high-throughput sequencing datasets of apple dormant bud, in vitro shoot, and cryopreservation-treated in vitro shoot (CP-treated) tissue samples.

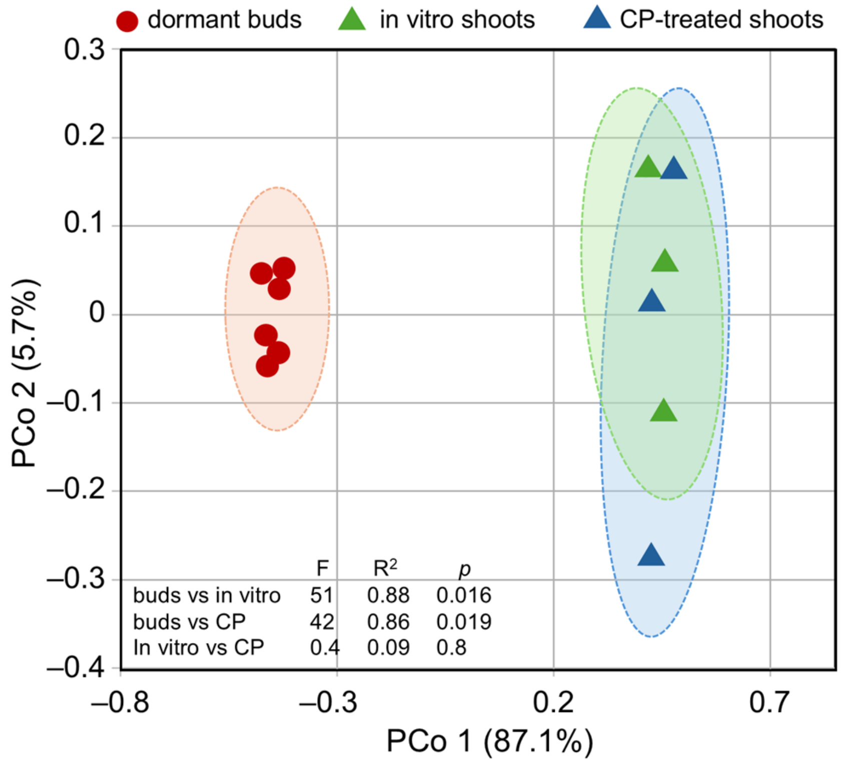

Figure S3. Bacterial diversity variation in apple tissue samples. The principal coordinate analysis (PCoA) of the ASV datasets generated using *16S rRNA* domain V4 amplicon high-throughput sequencing of dormant bud, in vitro shoot, and cryopreservation-treated in vitro shoot (CP-treated shoots) samples was carried out using the Bray–Curtis dissimilarity matrix.

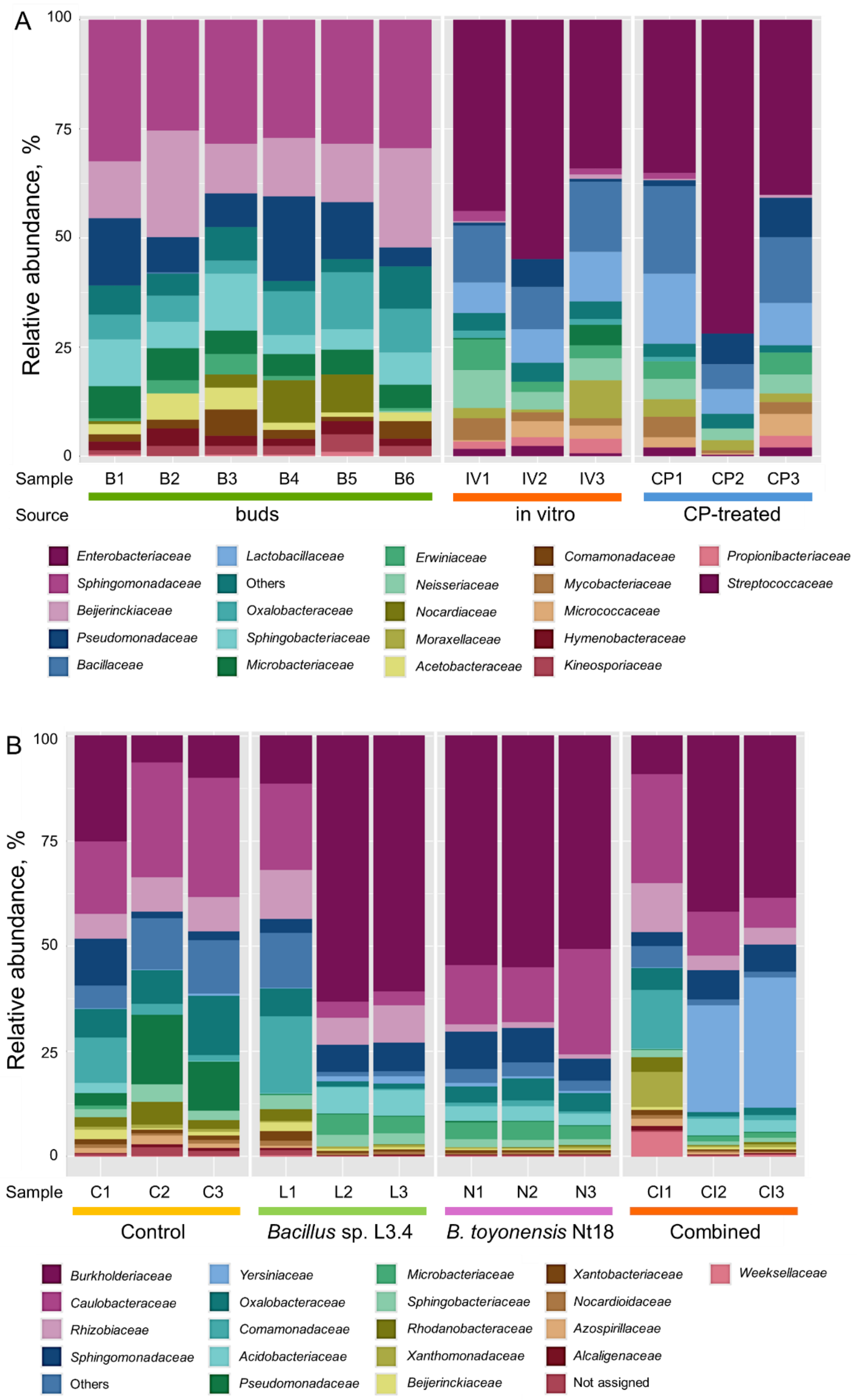

Figure S4. Composition and relative abundance of family-level endophytic bacterial taxa. The *16S rRNA* domain V4 amplicons of apple dormant bud, in vitro shoot, and cryopreservation-treated in vitro shoot (CP-treated) tissue samples (**A**) and control and bacterial inoculum-treated ex vitro acclimatised plantlet root samples (**B**) were analysed using high-throughput DNA sequencing.

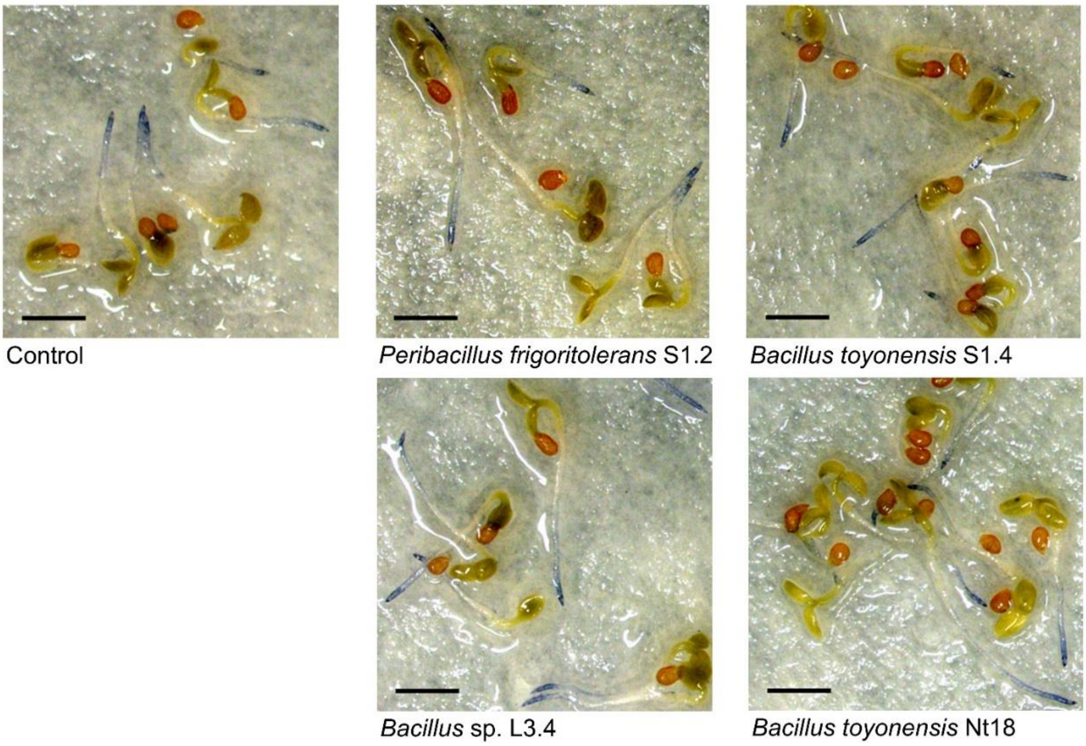

Figure S5. Tobacco seedling growth-modulating effect of selected endophytic bacterial isolates. Representative images of four endophytic bacterial isolates used in shoot rooting and ex vitro acclimatisation experiments are shown. The scale bar represents 1 mm.

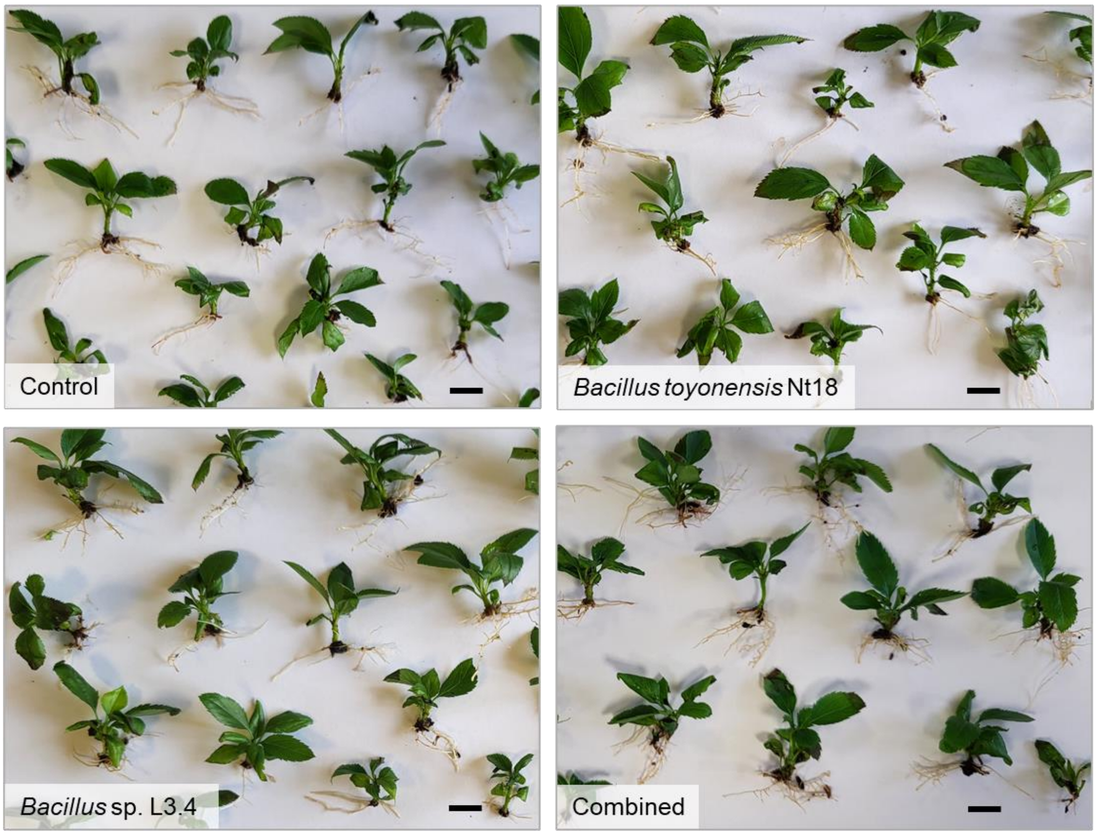

Figure S6. Representative images of control and bacterial inoculum-treated apple saplings after one-month of ex vitro acclimatisation. The scale bar represents 1 cm.

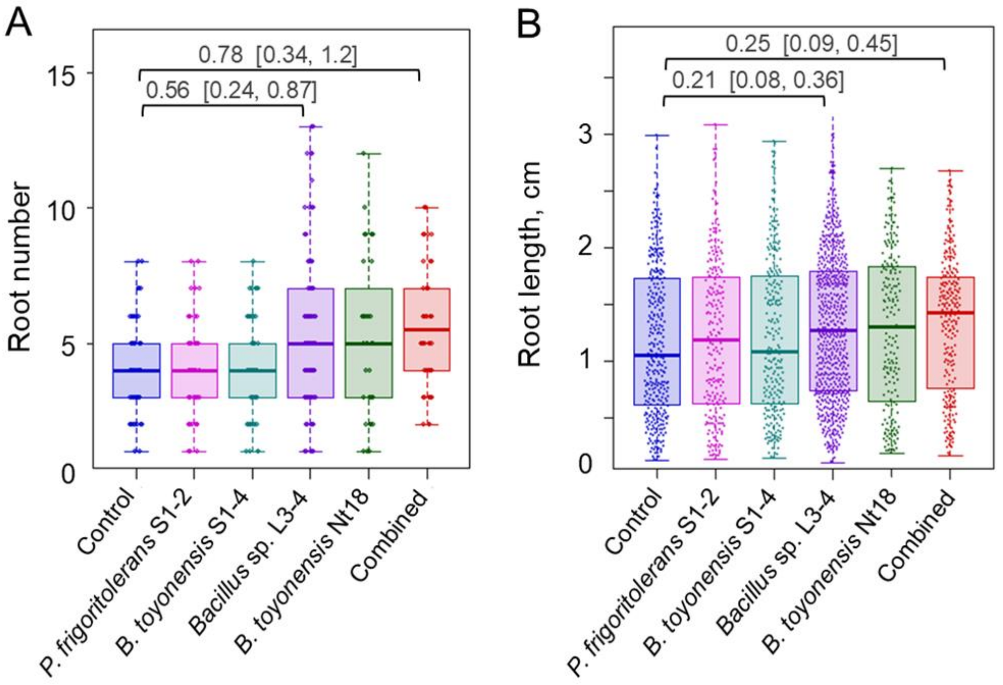

Figure S7. Endophytic bacteria inoculum effect on root number (**A**), individual root length (**B**) of ex vitro acclimatised apple plantlets. The data are shown as boxplots representing the medians, minimum and maximum scores, and lower and upper quartiles; data points are plotted as dots; numbers denote Hedge's g standardised mean difference effect size with a 95% confidence interval shown in the brackets.
